# Creep model of roadway surrounding rock considering hardening effect and damage effect

**DOI:** 10.1371/journal.pone.0331714

**Published:** 2025-09-29

**Authors:** Hongmiao Lv

**Affiliations:** School of Civil Engineering, Liaodong University, Dandong, China; Guizhou University, CHINA

## Abstract

The influence of confining pressure on creep characteristics was analyzed by carrying out creep tests of rocks under different confining pressures. A new creep model of roadway surrounding rock considering the combined influence of hardening effect and damage effect is established by introducing hardening function and damage variable. Combined with the yield function and plastic potential function, the one-dimensional sandstone creep model is extended to the three-dimensional sandstone creep model. Finally, the correctness of the model is verified by experimental data. The results show that the creep model curve of sandstone considering hardening and damage effects is in good agreement with the test curve. Only under the action of failure load, the model curve deviates from the test curve. But on the whole, the correlation coefficient between the two is above 0.90. This shows that the established model is correct. It also comprehensively reflects the common influence mechanism of hardening effect and damage effect of rock in the whole process of creep deformation. Compared with the Nishihara model, the model curve established in this paper better describes the creep curve with accelerated creep characteristics. At the same time, it also better describes the variation of decay creep and stable creep in creep behavior. When studying the influence of hardening effect and damage effect on rock creep behavior, it is necessary to comprehensively consider the combined effects of various influencing factors.In particular, the creep characteristics of different stages of accelerated creep, decay creep and stable creep are studied. Therefore, the established model can better simulate the characteristic behavior of different stages in the creep process of rock. It well reflects the influence of hardening and damage characteristics on the creep behavior of rock.

## 1 Introduction

In the deep mining environment, the mechanical environment of the surrounding rock of the roadway is extremely complex, showing the remarkable characteristics of ‘three high and one disturbance’ [1–2]. High ground stress makes the rock mass store huge energy, high osmotic pressure changes the physical and chemical properties of rock mass, high temperature affects the mechanical behavior of rock mass, and strong mining disturbance breaks the equilibrium state of original rock stress [3–4]. Under this complex condition, the surrounding rock of roadway shows a series of complex mechanical responses. After the excavation of the roadway, the original initial stress equilibrium state of the surrounding rock is completely broken. At the periphery of the roadway, the stress is redistributed, and the surrounding rock near the surface of the roadway first enters the plastic failure state, and the surface displacement of the surrounding rock of the roadway increases sharply. If effective support measures cannot be taken in time, the roadway is likely to deform and destabilize, which seriously threatens the safe production of the mine [5–7]. Different types of roadways, such as mining area roadways, mining roadways, development roadways, etc., have significant differences in the load conditions of surrounding rock. The roadway in the mining area is strongly affected by mining, and the mining roadway is subjected to the dynamic pressure brought by the advance of the working face during the mining process. The development roadway is mainly affected by the original rock stress and long-term geological tectonic stress.

Hardening effect and damage effect are important deformation mechanisms of rock in the loading process, and their influence on the creep characteristics of surrounding rock is multifaceted [8]. The hardening effect refers to the phenomenon that the deformation rate of rock increases with time after a period of deformation at a certain stress level. The hardening effect will cause the compressive strength of the surrounding rock to increase with the extension of the loading time, so that the creep rate of the surrounding rock gradually decreases, and the rate of creep deformation accumulation with time becomes slower [9]. The hardening effect of surrounding rock will affect its creep characteristics and increase the stability of surrounding rock. The damage effect refers to the process of micro-damage, micro-crack propagation and rock structure failure of rock under long-term stress [10]. The damage effect will lead to the gradual fatigue and failure of the internal structure of the surrounding rock, and weaken the overall compressive capacity and stability of the surrounding rock. The creep rate of surrounding rock increases, and the accumulation of creep deformation is accelerated, which affects the creep characteristics of surrounding rock [11].

In recent years, scholars both domestically and internationally have proposed a variety of constitutive models and analytical methods to study the hardening and damage effects of rocks. Siddiquee et al., [12] developed a time-dependent dual-hardening model to predict the behavior of soft rocks under compression and shear states. By introducing dual yield surfaces and Koiter’s generalization principle, they addressed the limitations of single-yield models in predicting volumetric deformation. Kamdem et al., [13] found that the hardening and softening processes of rocks occur more rapidly under higher confining pressures and proposed a variable fractional-order rheological model capable of effectively simulating the hardening and softening behaviors of rocks. Zhou et al., [14] proposed a rock constitutive model based on the subloading surface theory and a modified CWFS model, incorporating kinematic hardening, isotropic softening, and hardening effects. This model accurately describes the stress-strain curves, residual strain evolution, and confining pressure effects of rocks under triaxial cyclic loading. Zhang et al., [15] investigated the brittle characteristics and damage evolution of deep sandstone under hydro-mechanical coupling, proposing new brittleness evaluation indices and establishing a statistical damage evolution model that considers pore pressure effects. This model effectively describes the nonlinear hardening-softening and damage behaviors of porous rocks. Wang et al., [16] discovered through triaxial shear tests that the failure characteristics of soft rocks transition from strain softening to strain hardening as confining pressure increases. They proposed an improved Hoek-Brown strength criterion and a statistical damage constitutive model based on the Weibull distribution, which more accurately describes the damage evolution and stress-strain relationships of soft rocks. Kamdem et al., [17] further demonstrated that rocks soften more rapidly under high temperature and high stress conditions, while hardening is more pronounced under lower stress. Their proposed variable fractional-order rheological model effectively simulates the hardening and softening behaviors of rocks. These studies provide significant theoretical support for the long-term stability analysis and design of rocks in underground engineering.

The traditional creep model of roadway surrounding rock often only considers the influence of single factor on creep, and it is difficult to fully reflect the complex mechanical response of surrounding rock in practical engineering. However, in the actual situation, in the creep process of surrounding rock materials, the hardening effect and damage effect exist at the same time and influence each other. The hardening effect makes the strength of the surrounding rock material increase with the increase of deformation. The damage effect leads to the deterioration of the internal structure of the material and reduces its bearing capacity. Ignoring the combined effect of these two effects will lead to a large deviation in the prediction of creep behavior of surrounding rock. It cannot provide a reliable theoretical basis for engineering practice.

The rock creep model proposed in this study innovatively couples the hardening effect with the damage effect, breaking through the limitation of the traditional model that only considers a single mechanism. By introducing nonlinear strengthening parameters based on strain hardening theory, the strength improvement caused by internal structural reorganization of rock under stress is quantitatively described. At the same time, combined with the continuous damage mechanics framework, the dynamic evolution equation of crack propagation and deterioration is established. The weakening effect of microstructure damage on stress transfer and deformation rate is described. The synergistic characterization of the two can not only accurately capture the nonlinear characteristics of instantaneous elastic deformation, steady-state creep and accelerated failure stages during rock creep. It also reveals the regulation law of hardening and damage on creep mechanism. Compared with the existing models, this method shows higher fitting accuracy and physical significance in the prediction of rock deformation under high stress and complex geological conditions, and provides a more universal theoretical tool for the stability analysis of deep rock mass engineering. In view of this, it is of great theoretical and practical significance to study the creep model of roadway surrounding rock considering the combined effect of hardening effect and damage effect. By establishing a more realistic creep model, the deformation process of surrounding rock under long-term complex stress can be simulated more accurately. It provides a solid theoretical support for the rational design and effective maintenance of deep roadway engineering.

## 2 Creep test of rock under different confining pressure

### 2.1 Triaxial mechanical tests of rock under different confining pressures

**Fig 1:**
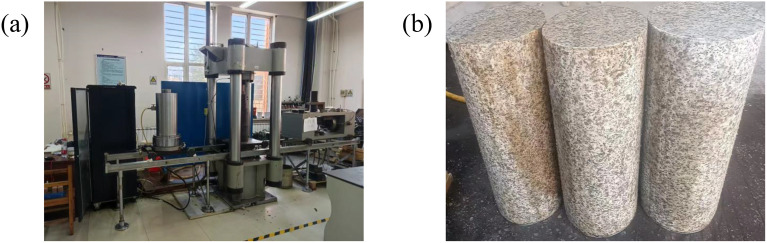
Rock testing machine and rock samples:(a)Rock testing machine,(b)rock samples.

The rock value is in the tunnel. The buried depth of the rock is about 500 m. The measured in-situ stress is between 10.12 MPa and 11.55 MPa. In order to study the influence of confining pressure on the creep characteristics of rock, the confining pressure values are set to 5MPa, 10MPa, 15MPa and 20MPa. The triaxial mechanical tests of rock under different confining pressures are carried out by using TAW-2000 rock testing machine(as shown in Fig 1(a)). The size of the rock samples are 100 mm in height and 50 mm in diameter(as shown in Fig 1(b)). The bulk density is generally 2400–2700 kg/m^3^, and the porosity is slightly higher than that of granite. Due to factors such as calcium carbonate mineral structure, it is roughly 2.5–3.0%. The main component is calcium carbonate (CaCO_3_), accounting for 65.12%. Containing a small amount of magnesium carbonate (CaMg (CO_3_)_2_), accounting for 8.87%. And trace silica (SiO2), accounting for 16.06%. iron oxide (Fe_2_O_3_), accounting for 6.85%. The remaining substances accounted for 3.10%.

The triaxial mechanical test steps of rock under different confining pressures are as follows.

(1) Representative rock samples are collected from the site. According to the relevant standards, the rock was processed into a cylinder specimen with a diameter of 50 mm and a height of 100 mm. The two ends of the specimen need to be smoothed to ensure that the non-parallelism error is within ± 0.05 mm. This ensures uniform stress during loading.
(2) A comprehensive inspection of the three-axis testing machine is carried out to ensure that the pressure control system, data acquisition system and other components can work normally. It is necessary to debug the equipment and set the confining pressure loading rate and axial loading rate. The pressure sensor and displacement sensor are calibrated.
(3) A thin layer of vaseline is evenly coated on the surface of the specimen to reduce the friction between the specimen and the pressure chamber. The prepared specimens are carefully installed in the pressure chamber of the triaxial testing machine, and the axial loading rod and the displacement measuring device are connected to ensure that the connecting parts are firm and not loose.
(4) It is necessary to close the pressure chamber and slowly inject hydraulic oil into the pressure chamber through the pressure control system. The confining pressures are applied to the target value according to the set loading rate. During the loading process, the pressure display instrument is closely observed to ensure the accuracy and stability of confining pressure loading.
(5) After the confining pressure is stabilized, the axial loading system is started to slowly increase the axial load at a predetermined loading rate. During the loading process, the data acquisition system records the axial pressure, axial displacement and confining pressure in real time. Continuous loadings are required until the specimen is destroyed, and the axial pressure and axial displacement data at the time of failure are recorded.
(6) After the specimen is destroyed, the axial load is slowly unloaded first, and then the confining pressure is gradually released. It is necessary to open the pressure chamber, remove the damaged specimens, and clean up the residual rock debris and liquid in the pressure chamber to prepare for the next set of tests.
(7) According to different confining pressure conditions, the test is repeated according to the above steps, and at least 3 specimens are tested under each confining pressure condition to improve the reliability and representativeness of the test results. Through the analysis of multiple sets of test data under different confining pressures, the influence of confining pressure on the triaxial mechanical properties of rock is studied.

**Fig 2:**
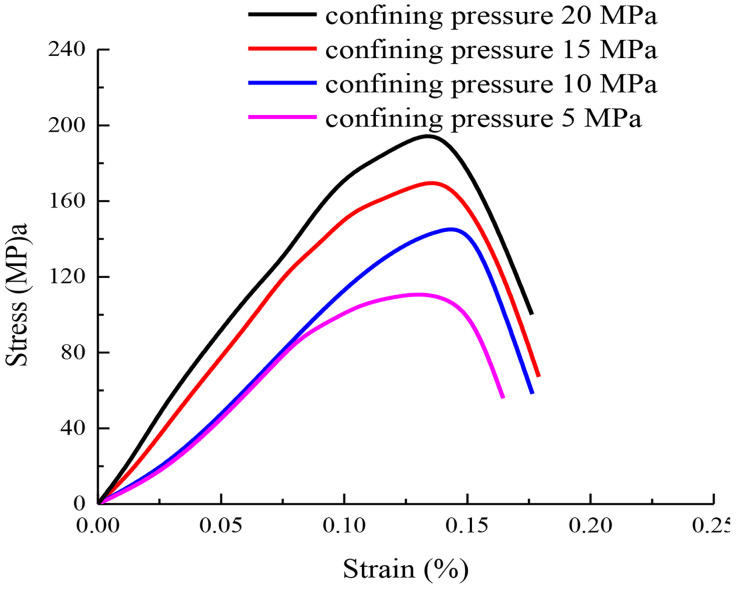
The triaxial stress-strain curves of rock under different confining pressures.

The triaxial stress-strain curves of rock under different confining pressures are drawn as shown in Fig 2.

It can be seen from Fig 2 that confining pressure has an important influence on the peak strength of rock, which is shown as follows. Under the condition of low confining pressure, the internal microcracks and cracks of rock are compressed by confining pressure. This effectively reduces the space and opportunity for crack propagation and increases the cohesion of the rock. Therefore, the existence of confining pressure can effectively improve the peak strength of rock. The existence of confining pressure changes the stress distribution and deformation mode inside the rock, which in turn affects the deformation characteristics of the rock. Under high confining pressure, the deformation capacity of rock is constrained. Therefore, the confining pressure has a great influence on the peak strength of rock when the stress is small. The existence of confining pressure is helpful to reduce the gap between rock particles and improve the density of rock. Therefore, the overall strength and stability of the rock are improved.

### 2.2 Triaxial creep test of rock under different confining pressure

According to the triaxial mechanical properties test, the triaxial creep test of rock under different confining pressures is drawn. The triaxial creep test steps of rock under different confining pressures are as follows.

(1) After the confining pressure is stable, the axial loading system is started, and the axial load is applied to the predetermined initial load value at a slower rate.
(2) After the initial axial load is applied, the load is kept constant and the creep monitoring stage is entered. The data acquisition system records the axial displacement, time and confining pressure in real time. During this period, the deformation of the specimen is closely observed. If abnormalities (such as displacement mutation) are found, the test and inspection equipment should be stopped in time.
(3) When the creep deformation rate of the specimen tends to be stable at a certain load level (for example, the change of deformation rate within 1 hour is less than 0.001 mm/ h), the axial load is increased according to a certain increment (such as 5% estimated compressive strength). The creep monitoring process needs to be repeated until the specimen is destroyed or reaches the maximum load predetermined by the test.
(4) When the test reaches the predetermined end condition (such as specimen failure or reaching the set maximum loading time), the axial load is slowly unloaded first, and then the confining pressure is gradually released. It is necessary to open the pressure chamber, remove the damaged specimens, and clean up the residual rock debris and liquid in the pressure chamber to prepare for the next set of tests.

**Fig 3:**
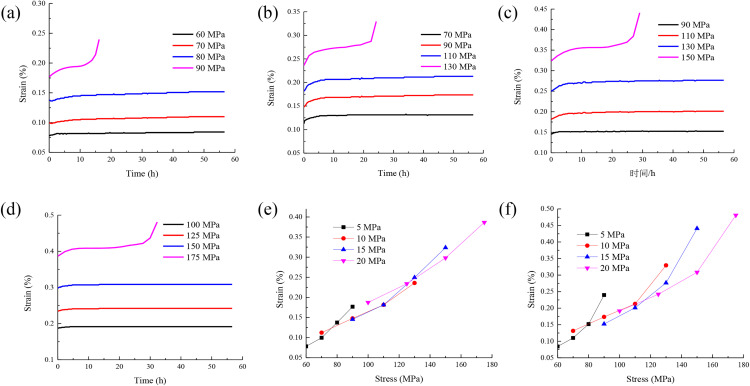
The triaxial creep curves of rock under different confining pressures:(a)the stress-strain curve of confining pressure 5MPa,(b)the stress-strain curve of confining pressure 10 MPa,(c)the stress-strain curve of confining pressure 15 MPa,(d)the stress-strain curve of confining pressure 20 MPa,(e)instantaneous strain,(f)creep deformation.

The triaxial creep curves of rock under different confining pressures are drawn as shown in Fig. 3.

It can be seen from Fig 3 that confining pressure and stress have an impact on the instantaneous strain and creep deformation of rock, which is mainly reflected in the following aspects.(1) The existence of confining pressure can slow down the deformation of rock during loading and reduce the instantaneous strain rate of rock. The increase of stress will lead to the increase of elastic strain of rock, which makes the instantaneous strain larger. Therefore, confining pressure and stress have opposite effects on the instantaneous strain of rock to a certain extent. (2) Confining pressure and stress also affect the creep deformation characteristics of rock. Confining pressure plays a positive role in the bearing capacity and stability of rock. (3) Confining pressure can improve the stability of rock, slow down the creep rate and reduce the strain rate of rock. The increase of stress may accelerate the deformation process of rock. This leads to larger instantaneous strain and creep rate increases. Therefore, in the design and prediction of rock engineering, it is necessary to comprehensively consider the dual effects of confining pressure and stress on the deformation behavior of rock to more accurately evaluate the stability and bearing capacity of rock.

With the increase of stress, the instantaneous strain and creep deformation of rock are increasing. This is because The rock shows elastic deformation at the initial stage of stress, and the stress is proportional to the strain (in accordance with Hooke’s law). When the stress increases, the mineral particles, crystal structure or micropores in the rock are compressed or stretched, and the lattice spacing changes reversibly, resulting in a linear increase of instantaneous elastic strain with stress.

(1) Viscoelastic deformation

Minerals (such as mica, clay minerals, etc.) in rocks will undergo slow slip of molecular chains or lattices under stress, showing creep deformation over time. The larger the stress is, the stronger the driving force to overcome the barrier between molecules is, the faster the viscous flow rate is, and the creep strain rate (such as the slope of the steady-state creep stage) increases significantly.

(2) Viscoplastic deformation

When the stress exceeds the long-term strength threshold of the rock, irreversible processes such as crystal dislocation motion and particle boundary slip are activated, resulting in viscoplastic creep. The higher the stress, the smaller the resistance of dislocation motion (such as the enhancement of thermal activation effect), the earlier the start time of accelerated creep stage, and the sharp increase of deformation.

**Fig 4:**
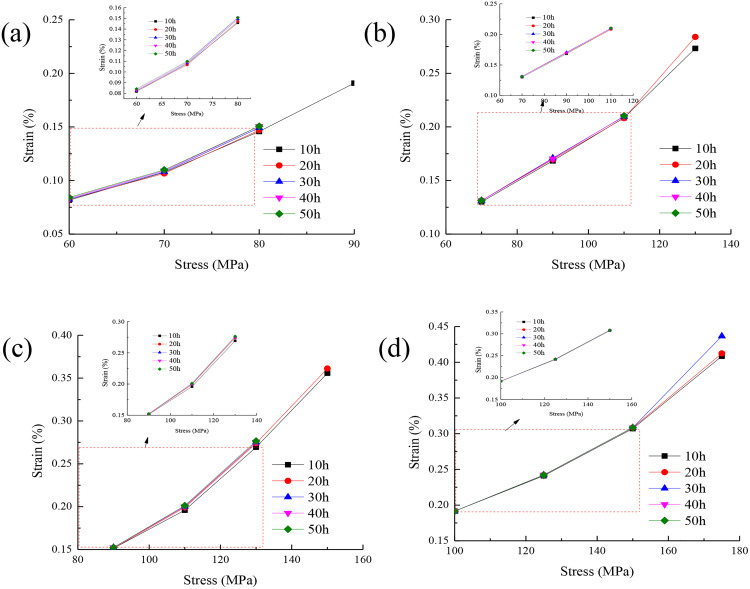
Isochronous stress-strain curves of rock under different confining pressures:(a)the isochronous stress-strain curve of confining pressure 5MPa,(b)the isochronous stress-strain curve of confining pressure 10 MPa,(c)the isochronous stress-strain curve of confining pressure 15 MPa,(d)the isochronous stress-strain curve of confining pressure 20 MPa.

The isochronous stress-strain curves of rock under different confining pressures are drawn as shown in Fig 4.

It can be seen from Fig 4 that from the isochronous stress-strain curve, it is observed that the curves under different stresses coincide before the stress of 80 MPa. This shows that the mechanical behavior of rock is relatively consistent in this range. When the stress reaches 80 MPa, the curve begins to diverge. This shows a significant difference. This phenomenon indicates that the rock exhibits good linear elastic properties in the low stress range. With the increase of stress, the nonlinear deformation characteristics of rock gradually appear. Therefore, the stress of 80 MPa can be used as the long-term strength of rock.

## 3 Creep model of rock considering hardening and damage effects

### 3.1 Rock creep model under one-dimensional stress state

(1) Establishment of hardening function and damage variable

Based on the theory of aging hardening function *H*(*σ*,*t*) can be expressed as


$$H\left(\sigma ,t\right)=C\sigma^{r}t^{1-\beta }$$ (1)

where *C*, *r* and *β* are material constants and *t* is time,*σ* is the stress.

In the process of loading creep, the internal structure of rock is continuously deteriorated due to stress and time, which leads to the deterioration of rock mechanical parameters and creep parameters. Therefore, this process is a process of gradual accumulation of damage. It can be assumed that the relationship between damage variables and time satisfies the following relationship [18].


D=1−exp(−αt) (2)

where *α* is the damage influence coefficient, *D* is the damage variable.

Therefore, the variable *Z* that comprehensively considers the hardening effect and damage effect of rock is


$$Z=H\left(1-D\right)=C\sigma^{r}t^{1-\beta }\cdot exp\left(-\alpha t\right)$$ (3)

(2) Establishment of instantaneous strain model

Since the instantaneous strain is only affected by stress and not affected by time. The instantaneous strain variable *Z*_*e*_ considering the hardening effect and damage effect of rock is


$$Z_{e}=H\left(1-D\right)=C_{e}\sigma^{r_{e}}\cdot exp\left(-\alpha_{e}\sigma \right)$$ (4)

where *C*_*e*_, *r*_*e*_ and *α*_*e*_ are material constants about instantaneous strain.

According to the generalized Hooke ‘s law and Eq. (4), we get


$$\varepsilon_{e}=\frac{\sigma }{E_{e}Z_{e}\left(\sigma \right)}=\frac{\sigma^{1-r_{e}}}{E_{e}C_{e}}$$ (5)

where *ε*_*e*_ is the instantaneous strain.

(3) Establishment of viscoelastic strain model

The hardening effect of the creep deformation stage is the main factor affecting the creep deformation law, but the model parameters also deteriorate under the action of time.


$$H_{ve}\left(\sigma ,t\right)=C_{ve}\sigma^{r_{ve}}t^{1-\beta_{ve}}$$ (6)

where *C*_*ve*_ and *r*_*ve*_ are constants about viscoelastic strain.

The viscoelastic strain damage variable *D*_*ve*_ is (Liu et al., 2024)


$$D_{ve}=1-exp\left[-\alpha_{ve}t\right]$$ (7)

where *α*_*ve*_ is the damage influence coefficient in the primary creep stage.

The instantaneous strain variable *Z*_*ve*_ considering the hardening effect and damage effect of rock is


$$Z_{ve}=H_{ve}\left(1-D_{ve}\right)=C_{ve}\sigma^{r_{ve}}t^{1-\beta_{ve}}\cdot exp\left(-\alpha_{ve}t\right)$$ (8)

The dashpot in the Kelvin model is replaced by the dashpot considering the hardening effect and the damage effect, and the following equation is obtained.


$$\sigma =E_{ve}\varepsilon_{ve}+\eta_{ve}\left(1-Z_{ve}\right)\varepsilon_{ve}^{\prime }$$ (9)

where *ε*_*ve*_ is viscoelastic strain, *ε*ʹ_*ve*_ is primary creep rate, *E*_*ve*_ is viscoelastic modulus, *η*_*ve*_ is viscoelastic viscosity coefficient.

By substituting Eq. (8) into Eq. (9), we obtain


$$\sigma =E_{ve}\varepsilon_{ve}+\eta_{ve}C_{ve}\sigma^{r_{ve}}t^{1-\beta_{ve}}\cdot exp\left(-\alpha_{ve}t\right)\varepsilon_{ve}^{\prime }$$ (10)

The initial condition is: when **t* *= 0, there exists *ε*_*ve*_ = 0. By integrating Eq. (10), we get


$$\varepsilon_{ve}=\frac{\sigma }{E_{ve}}\left\{1-exp\left[\frac{E_{ve}}{\eta_{ve}C_{ve}\sigma^{r_{ve}}}t^{\beta_{ve}}{\left(-\alpha_{ve}t\right)}^{-\beta_{ve}}\Gamma \left(\beta_{ve},-\alpha_{ve}t\right)\right]\right\}$$ (11)

where Γ(*a*,*x*) is an incomplete gamma function.

The incomplete gamma function Γ(*a*,*x*) can be expressed as


$$\Gamma \left(a,x\right)=\int_{0}^{\infty }t^{a-1}\exp\left(-t\right)dt$$ (12)

(4) Establishment of viscoplastic strain model

The viscoplastic hardening function *H*_*vp*_(*σ*,*t*) is


$$H_{vp}\left(\sigma ,t\right)=C_{vp}\sigma^{r_{vp}}t^{1-\beta_{vp}}$$ (14)

where *C*_*vp*_ and *r*_*vp*_ are constants with respect to viscoplastic strain.

The long-term strength *σ*_*s*_ of rock considering hardening effect is


$$\sigma_{s}=\sigma_{s0}+H_{vp}\left(\sigma ,t\right)$$ (15)

where *σ*_*s*0_ is the initial yield strength of rock.

Therefore, the viscoplastic model can be expressed as

When *σ* < *σ*_*s*_,


$$\varepsilon_{vp}=0$$ (16)

When *σ* ≥ *σ*_*s*_,


$$\sigma =\sigma_{s}+H_{vp}\left(\sigma ,t\right)+\eta_{vp}\frac{d\varepsilon_{vp}}{dt}$$ (17)

By substituting Eq. (15) into Eq. (17), we obtain


$$\sigma =\sigma_{s}+C_{vp}\sigma^{r_{vp}}t^{1-\beta_{vp}}+\eta_{vp}\frac{d\varepsilon_{vp}}{dt}$$ (18)

The initial condition is: when **t* *= 0, there exists *ε*_*vp*_ = 0. By integrating Eq. (18), we get


$$\varepsilon_{vp}=\frac{\sigma -\sigma_{s}}{\eta_{vp}}t+\frac{C_{vp}\sigma^{r_{vp}}}{\beta_{vp}-2}t^{2-\beta_{vp}}$$ (19)

(4) Establishment of accelerated creep model

In the process of rock creep deformation, the relationship between the damage variable *D*_*ac*_ of accelerated creep and time can be expressed as [19]


$$D_{ac}=1-\exp\left(-\alpha_{ac}t\right)$$ (20)

where *α*_*ac*_ is the damage influence coefficient of accelerated creep.

The hardening function *H*_*ac*_(*σ*,*t*) for accelerated creep is


$$H_{ac}\left(\sigma ,t\right)=C_{ac}\sigma^{r_{ac}}t^{1-\beta_{ac}}$$ (21)

where *C*_*ac*_ and *r*_*ac*_ are constants with respect to viscoplastic strain.

The instantaneous strain variable *Z*_*ac*_ considering the hardening effect and damage effect of rock is


$$Z_{ac}=H_{ac}\left(1-D_{ac}\right)=C_{ac}\sigma^{r_{ac}}t^{1-\beta_{ac}}\cdot exp\left(-\alpha_{ac}t\right)$$ (22)

Therefore, the accelerated creep model can be expressed as


$$\sigma =\eta_{ac}Z_{ac}\frac{d\varepsilon_{ac}}{dt}$$ (23)

By substituting Eq. (22) into Eq. (23), we obtain


$$\sigma =\eta_{ac}C_{ac}\sigma^{r_{ac}}t^{1-\beta_{ac}}\cdot exp\left(-\alpha_{ac}t\right)\frac{d\varepsilon_{ac}}{dt}$$ (24)

The initial condition is: when **t* *= 0, there exists *ε*_*ac*_ = 0. By integrating Eq. (24), we get


$$\varepsilon_{ac}=-\frac{\sigma }{\eta_{ac}C_{ac}\sigma^{r_{ac}}}t^{\beta_{ac}}{\left(-\alpha_{ac}t\right)}^{-\beta_{ac}}\Gamma \left(\beta_{ac},-\alpha_{ac}t\right)$$ (25)

The total strain *ε* under one-dimensional stress state is


$$\varepsilon =\varepsilon_{e}+\varepsilon_{ve}+\varepsilon_{vp}+\varepsilon_{ac}$$ (26)

By substituting Eqs. (5),(11),(16),(19) and (25) into Eq. (26), we obtain

When *σ* < *σ*_*s*_ and *t* < *t*_1_时,


$$\varepsilon =\frac{\sigma^{1-r_{e}}}{E_{e}C_{e}}+\frac{\sigma }{E_{ve}}\left\{1-exp\left[\frac{E_{ve}}{\eta_{ve}C_{ve}\sigma^{r_{ve}}}t^{\beta_{ve}}{\left(-\alpha_{ve}t\right)}^{-\beta_{ve}}\Gamma \left(\beta_{ve},-\alpha_{ve}t\right)\right]\right\}$$ (27)

When *σ* ≥ *σ*_*s*_ and *t* < *t*_1_时,


$$\varepsilon =\frac{\sigma^{1-r_{e}}}{E_{e}C_{e}}+\frac{\sigma }{E_{ve}}\left\{1-exp\left[\frac{E_{ve}}{\eta_{ve}C_{ve}\sigma^{r_{ve}}}t^{\beta_{ve}}{\left(-\alpha_{ve}t\right)}^{-\beta_{ve}}\Gamma \left(\beta_{ve},-\alpha_{ve}t\right)\right]\right\}+\frac{\sigma -\sigma_{s}}{\eta_{vp}}t+\frac{C_{vp}\sigma^{r_{vp}}}{\beta_{vp}-2}t^{2-\beta_{vp}}$$ (28)

When *σ* ≥ *σ*_*s*_ and *t* ≥ *t*_1_时,


$$\begin{array}{c} \varepsilon =\frac{\sigma^{1-r_{e}}}{E_{e}C_{e}}+\frac{\sigma }{E_{ve}}\left\{1-exp\left[\frac{E_{ve}}{\eta_{ve}C_{ve}\sigma^{r_{ve}}}t^{\beta_{ve}}{\left(-\alpha_{ve}t\right)}^{-\beta_{ve}}\Gamma \left(\beta_{ve},-\alpha_{ve}t\right)\right]\right\}\\ \;\;\;\;\;\;\;+\frac{\sigma -\sigma_{s}}{\eta_{vp}}t+\frac{C_{vp}\sigma^{r_{vp}}}{\beta_{vp}-2}t^{2-\beta_{vp}}-\frac{\sigma }{\eta_{ac}C_{ac}\sigma^{r_{ac}}}t^{\beta_{ac}}{\left(-\alpha_{ac}t\right)}^{-\beta_{ac}}\Gamma \left(\beta_{ac},-\alpha_{ac}t\right) \end{array}$$ (29)

**Fig 5:**
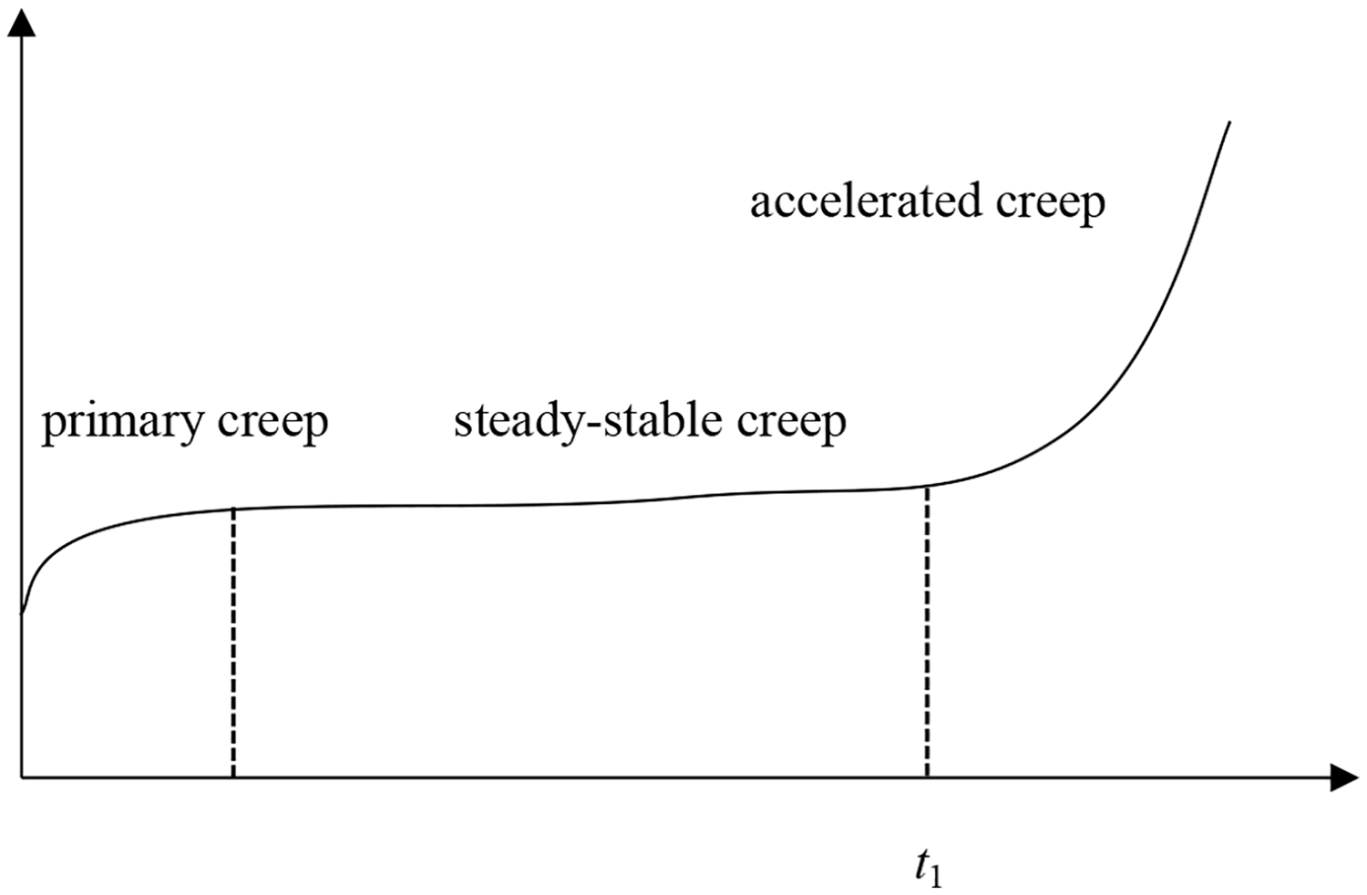
The determination method of time *t*_1._

The determination method of time *t*_1_ is shown in Fig 5.

It can be seen from Fig 5 that time t1 is the time corresponding to the boundary point of accelerated creep and steady-stable creep.

### 3.2 Creep model of rock under three-dimensional stress state

In practical engineering, the stress of the material is often three-dimensional and may be non-uniform. Only by establishing a creep model of three-dimensional stress state can the deformation and failure process of the material under complex stress state be accurately simulated, which provides accurate numerical simulation for in-depth understanding of material properties [20].

(1) Instantaneous strain under three-dimensional stress state

In a three-dimensional state, the strain and stress satisfy the following condition [21].


$$\left\{ \begin{array}{c} e_{ij}=\frac{\text{1}}{2G}S_{ij}\\ \varepsilon_{kk}=\frac{\text{1}}{3K}\sigma_{kk} \end{array}\right.$$ (30)

where *S*_*ij*_ is the stress partial tensor, *e*_*ij*_ is the strain partial tensor, *ε*_*kk*_ is the first invariant of the strain tensor, *σ*_*kk*_ is the first invariant of the stress tensor, *G* is the shear modulus, *K* is the bulk modulus.

According to Eqs. (5) and (30), we get


$$\varepsilon_{11}^{e}=\frac{{\left(\sigma_{1}+2\sigma_{3}\right)}^{1-r_{e}}}{9C_{e}K_{e}exp\left(-\alpha_{e}\sigma \right)}+\frac{{\left(\sigma_{1}-\sigma_{3}\right)}^{1-r_{e}}}{3C_{e}G_{e}exp\left(-\alpha_{e}\sigma \right)}$$ (31)

where *σ*_1_ is axial stress, *σ*_3_ is confining pressure, *K*_*e*_ is instantaneous bulk modulus, *G*_*e*_ is instantaneous shear modulus.

(2) Viscoelastic creep strain under three-dimensional stress state

According to Eqs. (11) and (30), we get


$$\varepsilon_{ve}=\frac{\sigma 1-\sigma 3}{3G_{ve}}\left\{1-exp\left[\frac{G_{ve}}{H_{ve}C_{ve}\sigma^{r_{ve}}}t^{\beta_{ve}}{\left(-\alpha_{ve}t\right)}^{-\beta_{ve}}\Gamma \left(\beta_{ve},-\alpha_{ve}t\right)\right]\right\}$$ (32)

where *H*_*ve*_ is the three-dimensional viscoelastic viscoplastic coefficient, and *G*_*ve*_ is the three-dimensional viscoelastic shear modulus.

(3) Viscoplastic creep strain under three-dimensional stress state

The yield function F is as follows.


$$F=\sqrt{J_{2}}-\sigma_{s}/\sqrt{3}$$ (33)

where *J*_2_ is the second invariant of the stress tensor.

When *σ* < *σ*_*s*_,


$$\varepsilon_{11}^{vp}=0$$ (34)

When *σ* ≥ *σ*_*s*_,


$$\varepsilon_{11}^{vp}=\frac{F}{2H_{vp}}t+\frac{C_{vp}F^{r_{vp}}}{\beta_{vp}-2}t^{2-\beta_{vp}}$$ (35)

where *H*_*vp*_ is the three-dimensional viscoplastic viscoplastic coefficient, *F* is the yield function.

(4) Accelerated creep strain under three-dimensional stress state

According to Eqs. (25) and (30), we get


$$\varepsilon_{11}^{ac}=-\frac{\sigma_{1}-\sigma_{3}}{3H_{ac}C_{ac}\sigma^{r_{ac}}}t^{\beta_{ac}}{\left(-\alpha_{ac}t\right)}^{-\beta_{ac}}\Gamma \left(\beta_{ac},-\alpha_{ac}t\right)$$ (37)

Therefore, the total strain of the three-dimensional stress state is


$$\varepsilon_{11}=\varepsilon_{11}^{e}+\varepsilon_{11}^{ve}+\varepsilon_{11}^{vp}+\varepsilon_{11}^{ac}$$ (38)

By substituting Eqs. (31),(32),(34),(35) and (37) into Eq. (38), we obtain

When *σ* < *σ*_*s*_ and *t* < *t*_1_时,


$$\begin{array}{c} \varepsilon_{11}=\frac{{\left(\sigma_{1}+2\sigma_{3}\right)}^{1-r_{e}}}{9C_{e}K_{e}exp\left(-\alpha_{e}\sigma \right)}+\frac{{\left(\sigma_{1}-\sigma_{3}\right)}^{1-r_{e}}}{3C_{e}G_{e}exp\left(-\alpha_{e}\sigma \right)}+\frac{\sigma 1-\sigma 3}{3G_{ve}}\cdot \\ \;\;\;\;\;\;\;\;\;\left\{1-exp\left[\frac{G_{ve}}{H_{ve}C_{ve}\sigma^{r_{ve}}}t^{\beta_{ve}}{\left(-\alpha_{ve}t\right)}^{-\beta_{ve}}\Gamma \left(\beta_{ve},-\alpha_{ve}t\right)\right]\right\} \end{array}$$ (39)

When *σ* ≥ *σ*_*s*_ and *t* < *t*_1_时,


$$\begin{array}{c} \varepsilon_{11}=\frac{{\left(\sigma_{1}+2\sigma_{3}\right)}^{1-r_{e}}}{9C_{e}K_{e}exp\left(-\alpha_{e}\sigma \right)}+\frac{{\left(\sigma_{1}-\sigma_{3}\right)}^{1-r_{e}}}{3C_{e}G_{e}exp\left(-\alpha_{e}\sigma \right)}+\frac{\sigma_{1}-\sigma_{3}-\sigma_{s}}{2\sqrt{3}H_{vp}}t+\frac{C_{vp}{\left(\frac{\sigma_{1}-\sigma_{3}}{\sqrt{3}}\right)}^{r_{vp}}}{\beta_{vp}-2}t^{2-\beta_{vp}}\\ \;\;\;\;\;\;\;\;\;\;+\frac{\sigma 1-\sigma 3}{3G_{ve}}\left\{1-exp\left[\frac{G_{ve}}{H_{ve}C_{ve}\sigma^{r_{ve}}}t^{\beta_{ve}}{\left(-\alpha_{ve}t\right)}^{-\beta_{ve}}\Gamma \left(\beta_{ve},-\alpha_{ve}t\right)\right]\right\} \end{array}$$ (40)

When *σ* ≥ *σ*_*s*_ and *t* ≥ *t*_1_时,


$$\begin{array}{c} \varepsilon_{11}=\frac{{\left(\sigma_{1}+2\sigma_{3}\right)}^{1-r_{e}}}{9C_{e}K_{e}exp\left(-\alpha_{e}\sigma \right)}+\frac{{\left(\sigma_{1}-\sigma_{3}\right)}^{1-r_{e}}}{3C_{e}G_{e}exp\left(-\alpha_{e}\sigma \right)}+\frac{\sigma_{1}-\sigma_{3}-\sigma_{s}}{2\sqrt{3}H_{vp}}t+\frac{C_{vp}{\left(\frac{\sigma_{1}-\sigma_{3}}{\sqrt{3}}\right)}^{r_{vp}}}{\beta_{vp}-2}t^{2-\beta_{vp}}\\ \;\;\;\;\;\;\;\;\;\;+\frac{\sigma 1-\sigma 3}{3G_{ve}}\left\{1-exp\left[\frac{G_{ve}}{H_{ve}C_{ve}\sigma^{r_{ve}}}t^{\beta_{ve}}{\left(-\alpha_{ve}t\right)}^{-\beta_{ve}}\Gamma \left(\beta_{ve},-\alpha_{ve}t\right)\right]\right\}-\frac{\sigma_{1}-\sigma_{3}}{3H_{ac}C_{ac}\sigma^{r_{ac}}}t^{\beta_{ac}}{\left(-\alpha_{ac}t\right)}^{-\beta_{ac}}\Gamma \left(\beta_{ac},-\alpha_{ac}t\right) \end{array}$$ (41)

In summary, the creep model of roadway surrounding rock considering the combined effect of hardening effect and damage effect is obtained.

## 4 Parameter identification and verification of rock creep model

Based on the software Origin and the least square method, the model parameters are identified, and the parameter values of the creep model of roadway surrounding rock considering the combined effect of hardening effect and damage effect are shown in Table 1. (Take the confining pressure of 5MPa as an example).

**Fig 6:**
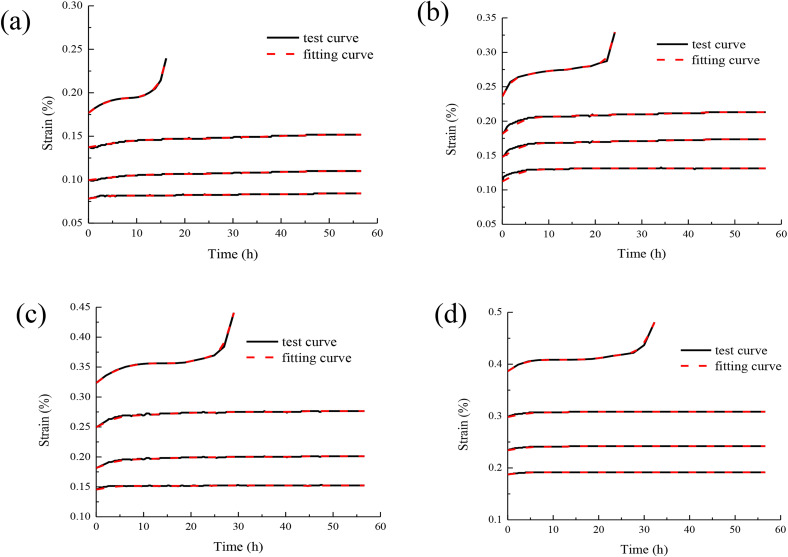
Comparison of sandstone creep model curves considering hardening and damage effects:(a)fitting curve of confining pressure 5MPa,(b)fitting curve of confining pressure 10 MPa,(c)fitting curve of confining pressure 15 MPa,(d)fitting curve of confining pressure 20 MPa.

The model parameters in Table 1 are substituted into the model, and the sandstone creep model curves considering hardening and damage effects are obtained, as shown in Fig 6.

**Table 1:** Creep model parameter values of roadway surrounding rock considering the combined effect of hardening effect and damage effect.

Stress/MPa	60	70	80	90
*K*_*e*_*/*GPa	18.277	15.235	12.579	10.538
*G*_*e*_/GPa	39.213	38.511	36.244	33.945
*H*_*ve*_*/*GPa·h	252.053	276.985	302.600	353.238
*G*_*ve*_*/*GPa	23.947	22.624	19.195	15.444
*H*_*vp*_/GPa·h	–	–	106.151	192.882
*H*_*ac*_/GPa·h	–	–	106.151	192.882
*r*_*e*_	0.274	0.207	0.187	0.172
*α*_*e*_	0.542	1.716	1.032	0.489
*r*_*ve*_	0.256	0.214	0.286	0.314
*α*_*ve*_	0.768	0.874	0.728	0.166
*r*_*vp*_	–	–	0.659	0.831
*β*_*ve*_	0.107	0.095	0.100	0.089
*β*_*vp*_	–	–	0.432	0.536
*α*_*ac*_	–	–	0.145	0.795
*C*_*e*_	0.469	0.365	0.268	0.152
*C*_*ve*_	0.313	0.381	0.387	0.441
*C*_*vp*_	–	–	0.462	0.665
*C*_*ac*_	–	–	–	0.667
*r*_*ac*_	–	–	–	−1.267
*β*_*ac*_	–	–	–	0.556
*R* ^2^	0.955	0.963	0.957	0.924

It can be seen from Fig 6 that the creep model curve of sandstone considering hardening and damage effects is in good agreement with the test curve. Only under the action of failure load, the model curve deviates from the test curve. But on the whole, the correlation coefficient between the two is above 0.90. This shows that the model established in this paper is correct and fully reflects the common influence mechanism of hardening effect and damage effect of rock in the whole process of creep deformation. In the study of creep behavior, it is very important to pay attention to the combined effect of hardening effect and damage effect. Because they jointly determine the deformation characteristics and mechanical properties of rock under continuous loading. The model can well reflect the creep behavior of rock on the basis of considering the hardening effect and damage effect. The accuracy and reliability of the model are verified by comparing with the experimental data. This provides strong support for in-depth understanding of rock creep mechanism and engineering applications. At the same time, the combined effects of hardening effect and damage effect on rock creep behavior are studied in depth. This is helpful to provide more scientific suggestions and guidance for rock engineering design and construction.

**Fig 7:**
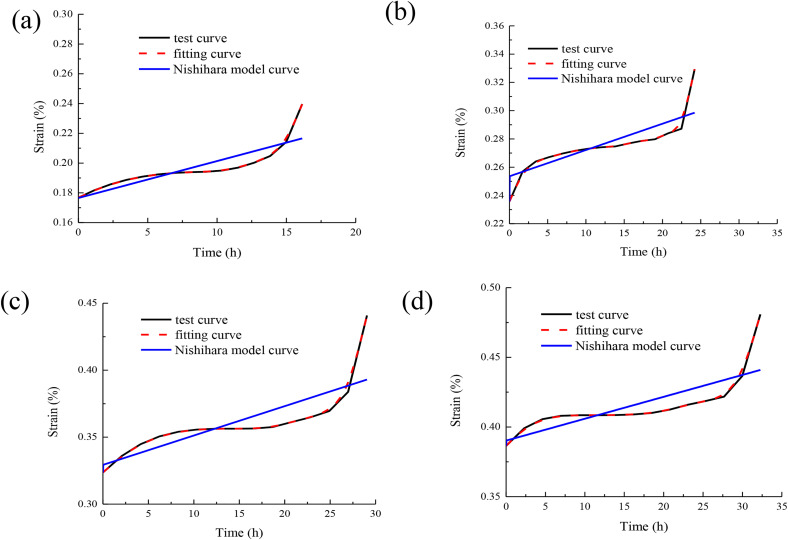
Comparison between Nishihara model and the model curve:(a)comparison curve of confining pressure 5MPa,(b)comparison curve of confining pressure 10 MPa,(c)comparison curve of confining pressure 15 MPa,(d)comparison curve of confining pressure 20 MPa.

In order to further highlight the correctness and superiority of the model, the Nishihara model is compared with the model (as shown in Fig 7).

According to the analysis results of Fig 7, compared with the Nishihara model, the model curve established in this paper better describes the creep curve with accelerated creep characteristics. At the same time, the model also better describes the variation of primary creep and steady-stable creep in creep behavior. This also shows that the model established in this paper more accurately reflects the evolution process of hardening and damage characteristics in rock creep. At the same time, when studying the influence of hardening effect and damage effect on rock creep behavior, it is necessary to comprehensively consider the combined effects of various influencing factors. In particular, the creep characteristics of different stages of accelerated creep, primary creep and steady-stable creep are studied. Therefore, the model established in this paper can better simulate the characteristic behavior of different stages in the creep process of rock, and better reflect the influence of hardening and damage characteristics on the creep behavior of rock.

Compared with the harding-damage coupling model proposed in this study, the classical Nishihara creep model shows poor matching when describing the deformation of surrounding rock in roadways. The root cause lies in the limitations of the model structure and mechanical mechanism. The Nishihara model is based on the traditional viscoelastic-viscoplastic series framework. Through the combination of Hooke body, Kelvin body and St. Venant body, although it can describe the instantaneous elastic deformation, steady-state creep and plastic flow processes of rocks, it does not consider the dynamic influence of stress state on the evolution of the internal structure of rocks. Specifically, there are the following two reasons.(1) The Nishihara model assumes that the viscosity coefficient is a constant, which cannot reflect the enhanced hardening effect caused by the rearrangement of rock particles and dislocation slip under high stress, as well as the damage deterioration caused by micro-fracture propagation. However, the model in this study can quantitatively describe the synergistic effect of hardening and damage by introducing strain hardening parameters and damage variables.(2) The Nishihara model’s prediction of the accelerated creep stage relies on a fixed plastic flow criterion, ignoring the nonlinear accelerating effect of damage accumulation on the creep rate of the surrounding rock of the roadway under complex stress paths such as excavation unloading and support constraints. Therefore, in the engineering environment of high stress and strong disturbance in deep roadways, the Nishihara model is unable to capture the dynamic coupling mechanism of hardening and damage. It can resulte in a significant deviation between its prediction results and the actual deformation.

In summary, the results of this study contribute to a deeper understanding of the causes and evolution of rock creep behavior, and provide a more accurate theoretical basis for the evaluation of rock stability and safety in rock engineering design and construction. At the same time, further exploration of the mechanism of hardening and damage characteristics in rock creep will help to improve the understanding of rock engineering problems. It provides more scientific and reliable guidance and suggestions for solving the problem of rock creep in practical engineering.

**Fig 8:**
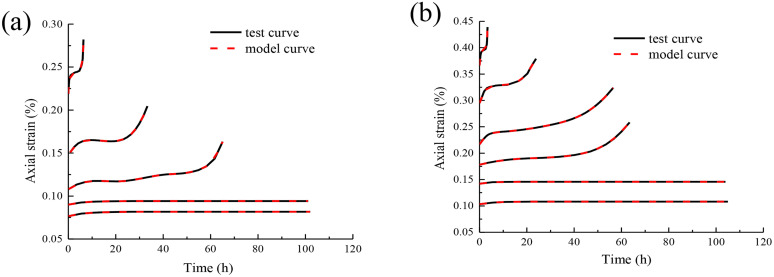
The comparison between different types of rock test curves and creep model curves in the literature:(a) confining pressure 10 MPa,(b) confining pressure 15 MPa.

The comparison between different types of rock test curves and creep model curves in the literature [22] are shown in Fig 8.

According to the analysis results of Fig 8, the creep test curves of different types of rocks are in good agreement with the established creep model curves, indicating that the model can effectively describe the creep deformation characteristics of various rocks under different stress conditions. The model parameters can be accurately inversed by experimental data, and have high fitting accuracy for the instantaneous elastic deformation, steady-state creep and accelerated creep stages of rock, which provides a reliable mathematical tool for the theoretical analysis of rock creep behavior. The results of good agreement show that although the mineral composition, structural characteristics and physical and mechanical properties of different rocks are different (such as the porosity and crystal structure of carbonate rocks and magmatic rocks are different), the creep deformation process still follows similar mechanical laws, that is, the creep deformation can be decomposed into the superposition of elastic, viscoelastic and viscoplastic components, which verifies the universality of creep theory in rock materials.

## 5 Conclusions

(1) The creep model curve of sandstone considering hardening and damage effects is in good agreement with the test curve. Only under the action of failure load, the model curve deviates from the test curve. But on the whole, the correlation coefficient between the two is above 0.90. This shows that the model established in this paper is correct and fully reflects the common influence mechanism of hardening effect and damage effect of rock in the whole process of creep deformation.
(2) Compared with the Nishihara model, the model curve established in this paper better describes the creep curve with accelerated creep characteristics, and also better describes the variation of decay creep and stable creep in creep behavior.
(3) When studying the influence of hardening effect and damage effect on the creep behavior of rock, it is necessary to comprehensively consider the combined effects of various influencing factors. In particular, the creep characteristics of different stages of accelerated creep, decay creep and stable creep are studied. Therefore, the model established in this paper can better simulate the characteristic behavior of different stages in the creep process of rock, and better reflect the influence of hardening and damage characteristics on the creep behavior of rock.
(4) Given that the creep model proposed in this study considering the coupling of hardening effect and damage effect has demonstrated a good explanatory ability for the deformation mechanism of surrounding rock in roadways at the theoretical level, subsequent studies can further deepen its engineering application value through discrete element numerical simulation [23]. In the future, the mesoscopic structure evolution of rocks (such as grain fragmentation and fracture propagation) can be cross-scale correlated with the macroscopic mechanical parameters of hardening and damage in this model. By introducing the dynamic degradation criterion of inter-particle bonding strength and the stress-strain hardening law into the discrete element framework, a numerical analysis platform that can truly reflect the progressive failure process of the surrounding rock of the roadway is constructed.
